# The Effect of Dexamethasone on Postoperative Pain in Patients After Laparoscopic Cholecystectomy

**DOI:** 10.7759/cureus.32067

**Published:** 2022-11-30

**Authors:** Kanwal Jamil, Rameez Qaisar

**Affiliations:** 1 Surgery, Russells Hall Hospital, Dudley, GBR; 2 Surgery, Benazir Bhutto Hospital, Rawalpindi, PAK

**Keywords:** visual analog score, postoperative pain management, analgesia, dexamethasone, laparoscopic cholecystectomy, pain, laparoscopy, cholecystectomy

## Abstract

Objectives

The objective of the study was to determine the effect of a single dose of IV dexamethasone on postoperative pain in patients after laparoscopic cholecystectomy. The outcome will be measured in the terms of mean pain score.

Study design and setting

This is a prospective study. We did a randomized control trial to compare the outcome in two groups. This study was conducted in the Department of Surgery, Benazir Bhutto Hospital, Rawalpindi, from December 2021 to May 2022. The total duration of the study was six months.

Methodology

A total of 160 patients were randomly divided into group A and group B. We performed laparoscopic cholecystectomies on all the patients under standard general anesthesia. In group A (control group), 5 mL of normal saline was injected intravenously at the time of induction of anesthesia. In group B, the dexamethasone group, the inj. dexamethasone with a dose of 0.1 mg/kg diluted in 5 mL normal saline was given intravenously at the time of induction of anesthesia. Postoperatively, the median pain score was measured using visual analog scale (VAS) at 2, 6, 12, and 24 h on a specially made proforma. The results were further stratified according to gender and age.

Results

The postoperative VAS in group B was significantly low compared with group A when measured at 2, 6, 12, and 24 h. It means that the median pain score was markedly less in the study group than in the placebo one, and it was statistically significant (p<0.05).

Conclusion

Administration of a single dose of dexamethasone preoperatively in laparoscopic cholecystectomy patients is effective to control postoperative pain.

## Introduction

Gallstones are present in up to 20% of the population, with their percentage slightly higher in females [1]. Gall bladder surgery is the accepted treatment option in such cases, whether by open or minimally invasive approach. However, recent advances have changed the paradigm over the last few decades, and now laparoscopic cholecystectomy (LC) is the main form of surgical intervention for cholelithiasis. Although it has the advantages of small-sized wounds, early recovery, and short hospital stays, it is not free of drawbacks [2]. Among its postoperative complications, postoperative pain is one of the biggest challenges health professionals face daily. This pain could be from a surgical site or the shoulder (referred pain).

There are various explanations to post LC pain. However, the most accepted reasons are peritoneal irritation due to carbon dioxide (CO_2_), peritoneal inflammation, and peritoneal stretching due to pneumoperitoneum. Medical practitioners have advised and used various techniques to minimize this, like preoperative administration of opioids or opioid-sparring analgesia, clonidine, preoperative pregabalin, wound site instillation of local anesthetics, and intraperitoneal irrigation with bupivacaine or ondansetron and suctioning of residual gas from the peritoneum before closure [3-10].

In this study, we planned to assess the effect of administering a single dose of dexamethasone before surgery on postoperative pain. Dexamethasone is a corticosteroid drug with excellent anti-inflammatory properties. By decreasing inflammation, it helps to control the pain [11]. In Pakistan, no such research work has been conducted. If we can prove the efficacy of dexamethasone in LC in our setup, then it would be beneficial for managing postoperative pain after LC.

## Materials and methods

In the Department of Surgery of Benazir Bhutto Hospital Rawalpindi, Pakistan, we conducted this study between December 2021 and May 2022. The sample size was calculated using the WHO sample size calculator keeping a level of significance of 5%, power of the test of 90%, population standard deviation (SD) of 1.94, test value of the population mean pain score of 4.03, and an anticipated population mean pain score of 3.03. A consecutive non-probability technique was used to select the patients while ensuring double-blinding. Patients of both genders aged 25-55 years undergoing elective laparoscopic cholecystectomy for cholelithiasis were included in this study. On the other hand, patients who were immunocompromised or had any chronic illness like diabetes mellitus (DM), ischemic heart disease (IHD), chronic kidney disease (CKD), chronic liver disease (CLD), any previous history of radiotherapy or chemotherapy, history of chronic pain of more than three months, any previous hepatobiliary surgery or who had laparoscopic cholecystectomy converted to open were excluded from this study.

Before surgery, we measured blood sugar levels (random) and other baseline investigations like complete blood count, blood urea/sugar, and hepatitis screening in addition to a preanesthesia workup. Cholelithiasis was confirmed on abdominal ultrasound. Formal approval was taken from the Research and Ethical Committee, Rawalpindi Medical University and Allied Hospitals, Rawalpindi with approval no. 294\IREF\RMU\2021. After approval, a total of 160 patients were admitted through the outpatient clinic of Benazir Bhutto Hospital Surgery Department as elective cases.

A total of 160 healthy and competent patients were recruited. The first author took thorough informed consent and then used concealed envelopes to allocate them to the two groups. The first author informed the anesthetists to administer the drug or placebo. Anesthesia notes were hidden from the patient and the assessors of pain who were the second author and the doctors of the surgical unit. All patients were given PO midazolam 7.5 mg orally the night before surgery. Appropriate intravenous antibiotic (inj. cefuroxime 1.5 g) prophylaxis was given to all patients within 60 min before the skin incision and another dose 6 h after surgery. All surgical procedures were performed under general anesthesia using endotracheal intubation (for controlled ventilation) and a combination of sevoflurane (inhalational anesthetic), propofol (IV anesthetic), fentanyl (analgesic), and atracurium (muscle relaxant). In group A (control group) 5 mL of normal saline was injected intravenously at the time of induction of anesthesia. In group B, the dexamethasone group, dexamethasone with a dose of 0.1 mg/kg diluted in 5 mL normal saline was given intravenously at the time of induction of anesthesia. After the surgery, the gas in the peritoneal cavity was thoroughly evacuated from the port sites. On the first postoperative day, all patients were given three doses of ketorolac 30 mg IV (analgesic) and Zantac 50 mg IV (H2 receptor blocker). All patients received the same level of care and were assessed for postoperative pain at 2, 6, 12, and 24 h by VAS. The on-call doctors assessed pain and documented it on the attached proforma in patients' notes along with the need for rescue analgesia. They gave the patients a dose of 75 mg of Dicloran IV as rescue analgesia when the pain score was >5 on VAS [12]. The proformas were unnamed and lacked patients' identities. Subsequently, these proformas were collected by the second author from the notes and then handed over to the first author who filled out the excel and SPSS sheets.

Data were analyzed using SPSS, version 21 (Armonk, NY: IBM Corp.). Means/medians and standard deviations were calculated for quantitative data like age and pain scores. We used frequency and percentage for the analysis of qualitative data like gender. VAS were compared between groups by independent sample t-test. Effect modifiers like age were controlled by stratification. Poststratification independent sample t-test was applied. A p-value <0.05 was considered statistically significant.

## Results

**Figure 1:**
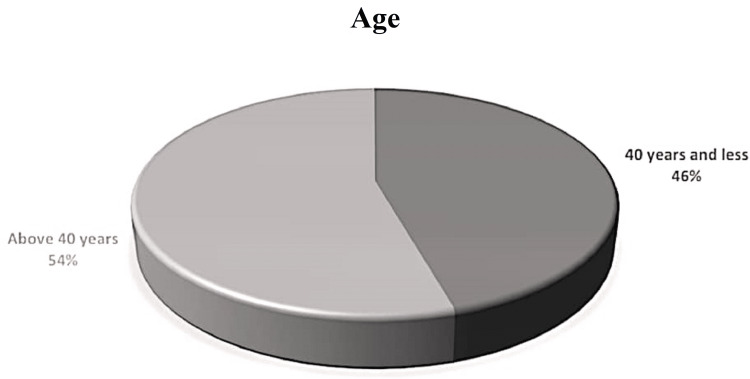
Age distribution after stratification.

**Figure 2:**
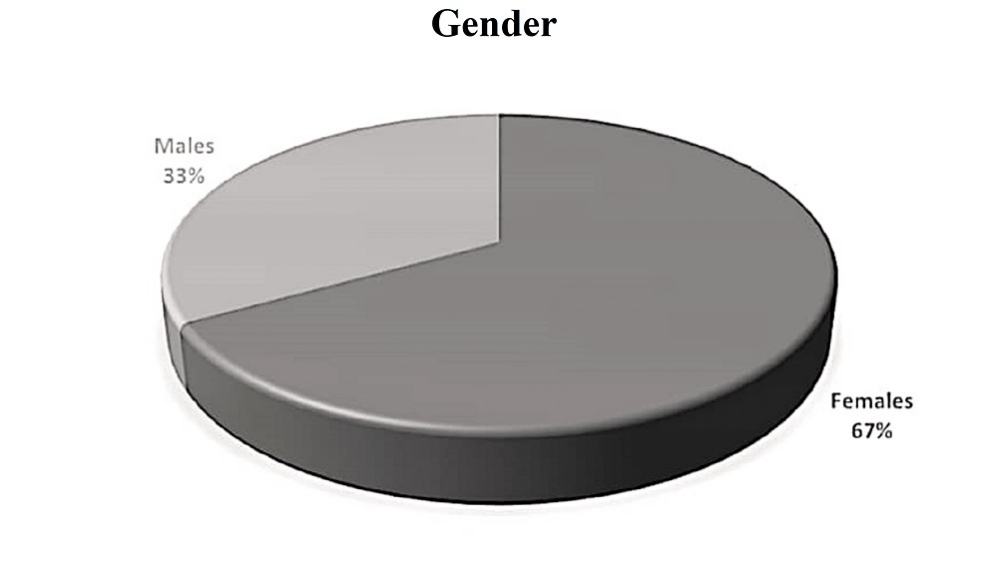
Gender distribution of the patients.

We included 160 patients in the study. The patients were divided into groups A and B, with 80 patients in each. The mean age of the patients was 40.38±9.59 years. The age range was 25-55 years. There were 66.9% (n=107) female and 33.1% (n=53) male patients. All the patients were stratified according to age group; 45.6% (n=73) patients were 40 years old or less, while 54.4% (n=87) patients were above 40 years of age. These distributions can be seen in Figures 1, 2.

We compared the median pain score between the two groups according to VAS at 2, 6, 12, and 24 h after the completion of surgery between the two groups. The results depicted a statistically significant difference in the pain score of the two groups at 6, 12, and 24 h after surgery. However, there was no significant difference in pain scores between the two groups, 2 h after surgery (p=0.054) (Table 1).

**Table 1:** Comparison of VAS between group A and group B at 2, 6, 12, and 24 h after surgery. VAS: visual analog scale

Median pain score	Group A	Group B	Significance (p-value)	t	SD group A/group B
VAS at 2 h	3 (mean=3.32)	2.5 (mean 2.86)	0.054	1.943	1.455/1.490
VAS at 6 h	4 (mean=4.18)	3.0 (mean=3.36)	0.000	3.565	1.509/1.371
VAS at 12 h	5 (mean=5.22)	3.0 (mean=3.56)	0.000	6.107	1.979/1.386
VAS at 24 h	3 (mean=3.71)	3.0 (mean=2.75)	0.001	3.311	2.119/1.480

The mean pain score was also compared by applying a t-test after stratification according to the age and gender of the patients. The age groups did not have a statistically significant difference but both genders had a statistically significant difference in pain scores at 2, 6, 12, and 24 h after surgery. The males had low VAS compared to females when assessed at each time interval. This is evident in Tables 2, 3.

**Table 2:** Comparison of VAS between different age groups at 2, 6, 12, and 24 h after surgery. VAS: visual analog scale

Median pain score	Age group 25-40 years	Age group 41-55 years	Significance (p-value)
VAS at 2 h	3 (mean=3.089)	3 (mean=3.088)	0.498
VAS at 6 h	4 (mean=3.884)	3 (mean=3.658)	0.169
VAS at 12 h	4 (mean=4.32)	4 (mean=4.47)	0.148
VAS at 24 h	3 (mean=3.115)	3 (mean=3.291)	0.276

**Table 3:** Comparison of VAS in both genders at 2, 6, 12, and 24 h after surgery. VAS: visual analog scale

Median pain score	Males	Females	Significance (p-value)
VAS at 2 h	2 (mean=2.51)	3 (mean=3.37)	0.000
VAS at 6 h	3 (mean=3.018)	4 (mean=4.149)	0.000
VAS at 12 h	3 (mean=4.17)	5 (mean=4.51)	0.016
VAS at 24 h	2 (mean=2.641)	3 (mean=3.55)	0.001

## Discussion

Laparoscopic cholecystectomy is the standard operative intervention for symptomatic cholelithiasis. The advantages of laparoscopic surgery over conventional open surgery are immense. This is due to less postoperative pain, shorter length of hospital stay, early return to the job, and decreased morbidity of the patients. Though there is an established superiority of laparoscopic cholecystectomy over open cholecystectomy, there are specific concerns about the procedure, with postoperative pain being the most important [13]. The type and nature of pain experienced by the patients after laparoscopic cholecystectomy is quite different from open surgery, and several studies are being done to overcome this significant concern.

In our study, the role of dexamethasone in reducing postoperative pain was evaluated. There are various studies present in the literature about the same subject. In a study done by Mohtadi et al., the role of dexamethasone in reducing postoperative pain was studied [14]. In that study, 122 patients were included, which is slightly lower than the sample size in our study (160 patients). The dose of dexamethasone is the same in both studies 0.1 mg/kg body weight of the patient given at the time of induction of anesthesia in one group. In contrast, the other group received placebo at the same time. Postoperative pain was studied according to VAS in both groups. The pain score at 2, 6, 12, and 24 h after surgery was determined to be significantly less in the dexamethasone group (p=0.003). These results validate the results of our study in which the mean pain score was also significantly less in the dexamethasone group of patients (p<0.001). Another factor studied in that study, not in our study, was the additional amount of analgesia required for the patients.

In another study by Lim et al., the role of dexamethasone in reducing postoperative pain and its timing of administration were studied [15]. There were three groups of patients in that study. Group N received a placebo, group S1 received an injection of 8 mg dexamethasone 1 h before surgery, and group S2 received the same dose of dexamethasone during laparoscopic cholecystectomy. There was statistically significant reduced pain in both S1 and S2 groups compared to group N (control group). However, there was no significant difference in pain scores between these two groups (S1 and S2). The results of this study also validate the results of our study that IV dexamethasone significantly reduces postoperative pain after laparoscopic cholecystectomy, and the timing of its administration does not affect its analgesic effects.

In a double-blinded trial conducted by Sánchez-Rodríguez PE et al., they measured the effect of IV dexamethasone against a placebo, given preoperatively to patients who were listed for laparoscopic cholecystectomy [16]. They measured the outcome in terms of postoperative nausea and vomiting (PONV), pain and fatigue scores. They inferred that the PONV and pain scores were significantly lower in the dexamethasone group than in the placebo. There was significantly less demand for ondansetron (p<0.001) and buprenorphine (p<0.009) to control PONV and pain, respectively. Similarly, Surender et al., in their trial successfully established that a single dose of dexamethasone given preoperatively can significantly improve the quality of recovery [17]. They compared the efficacy of diluted dexamethasone against diluted lignocaine given IV preoperatively. They measured the pain score and quality of recovery score (QoR-40). They found that dexamethasone is highly safe and effective to manage postoperative pain and promotes early emotional and physical recovery (p<0.001). The results of these trials further support our study and cement the efficacy of dexamethasone, which is not only effective in managing pain and PONV but also helps the patients to recover both emotionally and physically.

While most of the studies found in the literature affirm our study's results, a few studies show no significant difference in postoperative pain in the patients who were given dexamethasone preoperatively. A similar study was done by Gul et al. that showed that dexamethasone has a role in reducing postoperative pain, analgesia requirement, nausea, and vomiting [18]. However, these results were not statistically significant (p>0.05). This difference may be due to differences in the amount of dexamethasone (8 mg) given to all the patients irrespective of their weights and timing of administration (90 minutes before incision).

The role of dexamethasone is well-established in reducing postoperative pain in laparoscopic cholecystectomy as well as other laparoscopic surgeries. The results of our study further strengthen this fact. Additional benefits may include decreased postoperative nausea and vomiting.

There are a few limitations to our study. This study was conducted in a single center, and the sample size was small. Further, we have administered rescue analgesia at a VAS of 6 or more, whereas most literature indicates that rescue analgesia should be administered at VAS greater or equal to 4. It is likely to affect the total analgesia consumed. Our recommendations are to conduct this study on a larger scale to produce statistically more significant results to make this technique more useful in the future to control postoperative pain.

## Conclusions

In our study, we have successfully demonstrated that the administration of a single-dose injection of dexamethasone before induction of anesthesia significantly reduces postoperative pain after laparoscopic cholecystectomy. Good pain management leads to early recovery and short hospital stays which not just provide mental relaxation to hospital staff but also financial assistance to the trust. So, we recommend that dexamethasone is a safe and efficacious drug to reduce postoperative pain.
